# Energy Requirements of Beef Cattle: Current Energy Systems and Factors Influencing Energy Requirements for Maintenance

**DOI:** 10.3390/ani11061642

**Published:** 2021-06-01

**Authors:** Edward H. Cabezas-Garcia, Denise Lowe, Francis Lively

**Affiliations:** Sustainable Agri-Food Sciences Division, Agri-Food and Biosciences Institute (AFBI), Large Park, Hillsborough Co. Down BT26 6DR, UK; francis.lively@afbini.gov.uk

**Keywords:** energy requirements, beef cattle, feeding standards

## Abstract

**Simple Summary:**

The accurate estimation of energy requirements for present-day genotypes under current feeding conditions is crucial for improving profitability and reducing the environmental impact of the beef industry. Equations for predicting energy requirements of beef cattle according to the Agricultural and Food Research Council (AFRC) are outdated and require an urgent update. The results from literature review confirmed previous reports on the under prediction of energy requirements for maintenance by the AFRC, especially for growing animals. This may have consequences on the efficiency of use of the dietary energy on productive functions. Although much less research has been conducted over the last decade on energy metabolism for suckler cows, the existing data appears to be relevant as a valid reference for updating AFRC recommendations. The present review also revealed the lack of data on the contribution of both animal and diet-related factors influencing on energy requirements for beef cattle and thus conclusions on this regard are difficult to draw.

**Abstract:**

The present review compared features of the UK system for predicting energy requirements in beef cattle with a number of feeding systems developed from research institutes consortiums around the world. In addition, energy requirements for maintenance calculated from studies conducted at the Agri-Food and Biosciences Institute (AFBI) in Northern Ireland since the 1990s were compared with compiled data from recent peer-review papers published over the last decade (2009–2020). The mean metabolisable energy requirement for the maintenance (ME_m_) of growing cattle was 0.672 MJ/kg^0.75^ according to values obtained from calorimetry studies conducted at AFBI. This value is respectively 8.2 and 19.5% greater than the ME_m_ values obtained by the Agricultural and Food Research Council (AFRC), and the National Academies of Sciences, Engineering and Medicine (NASEM) equations, but it is in close agreement with the Institut National de la Recherche Agronomique (INRA) approach, when assuming a *Bos taurus* bull (300 kg LW) and an efficiency for converting energy for maintenance (*k_m_*) of 0.65. Most of the literature data on energy requirements for the maintenance for this animal category were obtained from studies conducted with *Bos indicus* animals and their crossbreds in Brazilian conditions with this confirming lower requirements of these animals when compared to pure *Bos taurus* cattle. A simulation of the total ME requirements calculated for an Angus × Friesian steer (LW = 416 kg) offered good quality grass silage, indicated that both AFRC and NASEM systems overestimate (38.5 and 20.5%, respectively) the observed efficiency of converting ME for growth (*k_g_*). When the total ME requirements (maintenance + growth) were assessed, both systems underpredicted total ME requirement in 15.8 and 22.1 MJ/d. The mean ME_m_ requirements for suckler cows obtained from the literature (0.596 MJ/kg^0.75^) is on average 19.1% greater than predictions given by both AFRC and INRA (lactation) equations when considering a 550 kg cow and a *k_m_* value of 0.72. Although no differences in net energy requirements for maintenance (NE_m_) were detected between dry and lactating suckler cows, as expected the later displayed greater variation as a result of differences in milk production. On this regard, the INRA model recognise increased NE_m_ requirements for lactating animals compared to dry cows. The re-evaluation of the concept of diet metabolisability and the analysis of existing data on compensatory growth responses are recommended for future updates of the British system (AFRC) having in to account the particularities of grass-based systems in the UK.

## 1. Introduction

Over a decade ago, a comprehensive review by Cottrill et al. [1] concluded that the Agricultural and Food Research Council (AFRC) recommendations [2] for feeding beef cattle in the United Kingdom (UK) were outdated and required an urgent revision. Energy intake is the most important factor affecting the growth rate and reproductive performance of beef cattle [3,4,5] and the accurate estimation of energy requirements for present-day genotypes under current feeding conditions is crucial for improving profitability and reducing environmental impact of the beef industry. One of the main concerns about using the AFRC system [2] today is the under-prediction of energy requirements for maintenance [1,6]. In line with this, changes in animal-related factors and feeding management practices over the course of last decades have contributed significantly to differences in terms of the efficiency of use of dietary energy for physiological functions. Dairy-origin growing and finishing beef cattle are now more common in the UK than four decades ago, and evidence from the literature supports an increased energy intake for these animals when compared with those of beef origin [7,8,9].

Although grass either grazed or conserved, is still the main and cheapest source of feed for beef cattle in the UK and Ireland, management practices have changed considerably since the latest version of AFRC [2] was released. Nowadays, high concentrate finishing diets for beef cattle are more widely used (although inputs are more expensive) and profitability relies more on improved feed efficiency (kg feed /kg animal product) [10]. In addition to both animal and diet-related factors contributing to outdated energy equations, confounding effects must be considered. For example, suckler cow equations in the UK system, were generated based on data taken from dairy cows without considering inherent particularities of this animal category. At present, societal concerns on the contribution of beef industry to climate change have increased considerably. In both UK and the Republic of Ireland, ruminants accounted for approximately 20% of all methane (CH_4_) emissions [11] and although it is well known that enteric CH_4_ is an energetic loss related the efficiency of the dietary energy by the animal, current AFRC recommendations did not include equations to predict it [2]. The objectives of this review were to describe and compare principles within AFRC equations and other feeding systems that are currently in use worldwide to predict energy requirements in beef cattle and to compare calculated energy requirements for maintenance in beef cattle based on results from studies conducted at the Agri-Food and Biosciences Institute (AFBI) since the 1990s and those published in recent peer-reviewed literature.

## 2. Materials and Methods

### 2.1. Description of Feeding Systems Predicting Energy Requirements of Beef Cattle

In the present review, equations are presented for predicting energy requirements for the maintenance and production functions (i.e., growth, gestation and lactation) developed from five research institute consortiums around the world including the Agriculture and Food Research Council (AFRC, [2]) in the UK; Commonwealth Scientific and Industrial Research Organisation (CSIRO, [12]) in Australia; Institut National de la Recherche Agronomique (INRA, [5]) in France; the National Academies of Sciences, Engineering and Medicine (NASEM, [13]) in North America and the Nutrient Requirements of Zebu and Crossbred Cattle (BR-Corte, [14]) in Brazil. All of these were developed by considering the particularities of each local beef industry context. Grass either grazed or conserved is usually the main forage source in the UK, Ireland and continental Europe. In these countries, grass silages can be partially or completely replaced with legumes (i.e., red clover) and whole-crop silages (barley, wheat, maize). In the UK, arable regions tend to utilise more concentrate feeds than traditional grass-based system. However, in typical grazing systems in Northern Ireland, the concentrate supplementation is seldom greater than 50% of the total diet (on a DM basis) during the growing phase and tends to increase at the finishing stage. Conversely, North American diets are typically characterised by greater concentrate proportion in the diet compared to the European diets. In the USA and Canada, the use of agricultural by-products (such as distillers’ grains) in the diets is more common than in Europe. Roughages such as maize silage, lucerne silage and hay are the main forage source in North America. In tropical areas of Australia and Brazil where energy systems have been developed, both feedlot and pasture on tropical grasslands feeding systems are present. Tropical forages are usually lower in protein contents and fibre digestibility compared to temperate species, which constrains animal performance [15,16].

The main aspects for predicting energy requirements according to these international models were discussed and simple comparisons were provided to assess the implications of predicting energy requirements in beef cattle depending on the choice of a particular feeding system. The relationships between live weight (LW) of *Bos taurus* bulls and suckler cows and ME_m_ calculated from equations within three energy systems [2,5,13] were presented graphically using the equations in Table A2 and Table A4 in the Appendix A.

### 2.2. Data Collection of Papers Determining Energy Requirements for Maintenance

Since feed energy required for beef cattle is first prioritised to meet their requirements for maintenance over production functions, the present review explored this by collecting data from scientific papers published in peer-review journals. A historical perspective on how energy maintenance requirements of beef cattle have changed since the last version of the AFRC [2] system was released (three decades ago), is given by the values (mean and s.d.) showed in the present literature review (2009–2020) and the summary (1989–2009) of values reported in an earlier literature review by Cottrill et al. [1]. The results from studies conducted at AFBI (1990–2020) which have been already published in peer-review journals, were considered as representative of today’s beef cattle production systems in the UK.

The database search included papers reporting energy requirements for maintenance for both growing animals and suckler cows. Data were obtained by searching a range of databases: CAB Abstracts, Web of Science, ISI Proceedings, BIOSIS Previews, Food Science and Technology Abstracts and MEDLINE. Up to 554 publications were retrieved using search terms including (keywords): energy requirements for maintenance, beef cattle, growing animals and suckler cows.

### 2.3. Inclusion Criteria

The study selection criteria were: (1) publication in English in a peer-reviewed journal, (2) energy requirements for maintenance (either on a metabolisable or net energy basis; ME or NE respectively) calculated based on one of the following methodologies: Calorimetry (using respiration chambers), comparative slaughter or long-term feeding trials. In occasions, the results derived meta-analysis studies compiling data from one of the referred methodologies were also included in the present review when relevant, (3) when the animal gender was reported or in the case of suckler cows the physiological state (i.e., pregnancy, lactation) was known, (4) breed type was not restrictive as much as it was mentioned in the original publication (this includes both *Bos taurus* and *Bos indicus* animals with their respective crossbreds) and (5) mean animal LW was available. All data was converted to mega joules (MJ) and requirements expressed in MJ/kg LW^0.75^ for comparison purposes among studies.

The study exclusion criteria were: (1) when energy requirements for maintenance were not obtained following one of the methodologies mentioned above. For example, studies involving head hoods since that is not a traditional method to estimate energy requirements in the feeding systems considered in the present review, (2) only peer-reviewed publications were included in the study, because the peer review process is a proxy for assessing the quality of studies [17]. Of the total of papers that were retrieved, only those that satisfied the predetermined inclusion criteria were finally included in the present review.

### 2.4. Data Analysis

Data collected on energy requirements for maintenance (either ME_m_ or NE_m_ basis) from individual studies were compiled in a Microsoft Excel spreadsheet (Microsoft Corp., Redmond, WA, USA). Descriptive statistics (mean and standard deviation) were calculated using PROC UNIVARIATE of SAS, version 9.4 (SAS Institute, Cary, NC, USA). When ME_m_ requirements were not reported in the original publication, an estimate of it was calculated from NE_m_ by taking the ratio NE_m_ to ME_m_, which is assumed to be *k_m_*. Otherwise, when possible, the latter was calculated from *k_m_* equations within proper feeding systems according to the production system context of a specific study. For growing animals in occasions, maintenance energy requirements were derived from the linear regression of energy retention in carcass (growth) against ME intake or heat production (HP) against ME intake. Depending on the data availability, additional box plots were considered in order to assess variability within animal category, measurement technique, etc.

## 3. Results and Discussion

### 3.1. An Overview of the AFRC System Compared to Other Feeding Standards around the World

A general overview of nutritional models currently used around the world for predicting energy requirements in beef cattle is presented in Table 1. Energy systems for growing beef cattle developed by the AFRC [2], CSIRO [12] and INRA [5] are based on calorimetry, whereas systems used in North America [6,13] and Brazil [14] rely on comparative slaughter trials. However, it is interesting to note that energy feeding systems for dairy cattle in the USA (which may be relevant for suckler cows) were developed from calorimetric data [18]. According to NRC [19], one limitation of using calorimetry estimates relates to their lack of applicability in practical feeding conditions.

The UK metabolisable energy (ME) feeding system based on calorimetry studies, was first proposed for use in the UK in 1965 by the Agricultural Research Council [20] to overcome deficiencies of the Starch Equivalent (SE) system (a net energy (NE) system) such as the assumption of a simple ratio of NE values of feeds for maintenance, fattening and lactation; in addition, the SE system did not account for the effect of feeding level on NE concentration of a feed [1]. The original ME system [3] was then simplified [21], revised [3] and further improved by The Agricultural and Food Research Council [22]. An advisory manual on energy and protein requirements of ruminants (dairy, beef, sheep, and goats) was finally released during the early 90s [2].

Calorimetry is the measurement of heat production. Energy contained in the feed (gross energy; GE) is not fully used by the animal since there are considerable losses associated with the digestion and metabolism of the nutrients in that feed occur. The ME is calculated by subtracting faecal, urinary and methane losses from the GE intake. Direct calorimetry is based on the same general principle as the bomb calorimeter, in that the heat evolved is used to increase the temperature of a surrounding medium; whereas indirect calorimetry is based on the relationship between the amount of heat produced for oxidation of food or body components and the amount of oxygen consumed, carbon dioxide produced, and nitrogen excreted in the urine [23]. Because direct calorimetry is difficult in practice, indirect calorimetry is usually preferred.

The heat expenditure (fasting heat production (FHP) plus fasting urinary output) obtained during fasting is the amount the animal uses for maintenance (i.e., NE_m_) and the heat expenditure (heat production) during the restricted feeding with zero energy for production is taken as ME_m._ due to the difficulties associated with estimating energy balance of animals offered diets at maintenance level and the influence of variables such as plane of nutrition, production level, visceral organ mass, breed and sex of animals and duration of measurement [23,24]. The ME_m_ is usually estimated either from fasting metabolism divided by the energetic efficiency for maintenance (*k_m_*) or from regression of energy intake against energy outputs [23]. A practical limitation of the ME system is that it is based on experiments using castrated male sheep rather than cattle. However, it would seem that differences between the two species in terms utilisation of energy were not that big [25]. Although calorimetry studies are expensive, labour-intensive and not specially designed for measuring a large number of animals simultaneously, these studies have been performed for over 100 years and provide the basis for our current understanding of energy metabolism in farm animals [26]. Animal energetics principles developed by the UK system [2] are widely shared across energy systems worldwide and the current Australian system for determining energy requirements in beef cattle. However, it is clear that changes in both dairy and beef industry (animals and diets) over the last four decades, have contributed to outdated energy requirements provided by the AFRC [2]. Modelling efforts during early the 2000s supported on evidence collected from individual studies, were focused on updating nutritional requirements for specialised dairy cattle and this was translated into the ‘Feed into Milk’ system (FIM), published in 2004 [27].

Comparative slaughter for determining energy requirements in beef cattle is based on The California Net Energy System published by Lofgreen and Garrett [28] in 1968. The most significant factor affecting maintenance requirements is LW, which is used primarily to estimate maintenance requirement (NE_m_). However, expressing energy requirements either in terms of shrunk body weight (SBW) or empty body weight (EBW) is preferred. In contrast to calorimetry, in which ME intake and heat energy (HE) are measured and retained energy (RE) is determined by difference, in comparative slaughter procedures ME and RE are measured directly and HE calculated by difference. In a growing animal, the RE is the NE required for gain (NE_g_), and the slope of the linear regression of RE on ME intake provides an estimate of the efficiency of utilisation of ME for RE (*k_g_*) [13]. The ME intake at which RE is equal to zero provides an estimate of ME required for maintenance (ME_m_), and the intercept of the regression of log HE on ME intake yields an estimate of FHP, which equates to NE_m_ [13]. Finally, the ratio NE_m_ to ME_m_ is assumed to be the efficiency of the utilisation of ME for maintenance (*k_m_*). Whilst comparative slaughter technique may allow for a better replication of production conditions when compared to calorimetry trials, it requires studies to be conducted over extended time periods to obtain accurate measures and is thus costly and labour consuming. One practical limitation of using energy equations by NASEM [13] is that these cannot be applied with confidence to cattle under 250 kg (pre-weaning phase).

In addition to both calorimetry and comparative slaughter methodologies, long-term feeding trials have been also used for estimating maintenance requirements by measuring the quantity of feed that will maintain a constant LW [29,30,31]. However, this is an approximation rather than an exact measure of maintenance requirements due to errors associated with difficulties related to the precision of LW measures, changes in gut fill and problems in defining exactly the total quantity of feed digested during the period of the study. Despite these considerations, long-term specially designed production studies trials are highly valuable to adjust energy requirements when there is little information available in the literature on the effects of animal, diet and management-related factors. An example of this has been successfully used for dairy cattle within the FIM system [27], a revised update of the previous AFRC guidelines for dairy cows [2].

There is no difference in principle between the ME (AFRC, CSIRO) and NE (INRA, NASEM, BR-Corte) systems, with both systems recognising that the energy requirement of ruminant animals is the sum of their energy requirements for maintenance, production (milk, LW gain and wool growth) and foetal growth [23]. For feed evaluation, both ME [2,12] and NE systems [5,13,14], still use ME concentration in feeds as the basic energy term for calculations. Net energy concentration in feeds cannot be measured, but estimated using its ME concentration multiplied by different energetic efficiencies *(k_s_*) depending on the animal production function (maintenance, NE_m_; LW gain, NE_g_; etc.). In the NE systems, a single feed can have different NE values depending on the functions of animal production, whereas in the ME systems, energy contents of feedstuffs are expressed as a single value in terms of ME units [23]. It is interesting to note that equations to predict enteric methane (CH_4_) production have been included in NE systems over the last decade [5,13,14] as a result of increased concern on the effect of the beef industry on climate change (Table 1). All CH_4_ equations consider dry matter intake (DMI) as the main driver to quantify total CH_4_ production (g/d), and both INRA [5] and NASEM [13] developed specific equations for low and high concentrate proportion in the diet by adding nutrient composition variables (such as digestible organic matter (DOM), neutral detergent fibre (NDF) and crude protein (CP), etc., and LW.

As expected, energy systems developed in tropical environments [12,14] have included specific equations or adjustments for predicting energy requirements in Zebu cattle and their crossbreds. On the other hand, the distinction between dairy and beef origin genotypes is considered in the French system in temperate regions [5]. Two major issues were raised by the NASEM [13] and deserve more attention in the future by different feeding standards worldwide. The first issue is related to the prediction of energy requirement for maintenance for grazing animals and the second issue is the calculation of energy required for animals under cold-stress conditions.

It is worth nothing that all systems described above have used a factorial approach (additive) to estimating energy requirements in beef cattle. Criticisms to these models are particularly addressed to the inadequate description of the interactions between feeds or nutrients, or the effect of these on the composition of animal products. The Ruminant Nutrition System [RNS, 6] is a recent further development of the Cornell Net Carbohydrate and Protein System (CNCPS), originally published by Fox et al. [32] and Tylutki et al. [33] aiming for a better understanding of animal energetics by a more mechanistic approach. From a factorial approach perspective, principles for determining energy requirements for beef cattle using the RNS [6] are rather similar to those in NASEM [13]. Because of that, it was decided to exclude the features of the RNS system from the present review. In addition, a direct comparison of mechanistic approaches (level 2) by RNS with factorial approaches within energy systems included here, is not an easy and straightforward task. However, importance of the theoretical background proposed by the RNS system [6] is recognised for future improvements of the current UK system. Even though there are a number of feeding systems across Europe which are applicable at some extent to the UK conditions, the French system was chosen for comparison purposes mainly due to the potential relevance of their more recent updates [5]. Despite the tropical conditions where the Brazilian system [14] was originally developed, this system is included in the present review mainly due to its high impact in the research on beef cattle energetics, specially over the last decade (2009–2020).

### 3.2. Energy Efficiencies

The equations used to calculate the efficiencies of ME use for maintenance, LW gain and lactation are presented in Table A1 in the Appendix A. All energy systems reported a higher efficiency of ME utilisation for maintenance than for productive functions (i.e., LW gain or fattening). The equation for predicting *k_m_* according to the French model [5] leads to an increased efficiency when compared to the British and American models [2,13]. Both AFRC [2] and CSIRO [12] ME systems recognise an increased efficiency for lactation compared to LW gain. It is worth noting from Table A1 that it is not possible to establish a direct comparison in terms of energy efficiency of lactation between the AFRC based models [2,12] and the INRA [5], since the last system combines the efficiency for maintenance and milk yield in a single parameter (*k_ls_*).

Among systems [2,5,12,13], calculated efficiencies for both maintenance and productive functions increase with increasing dietary energy metabolisability (ME/GE). In practice, this criterion is closely related to the digestibility of the organic matter (OM) which is also a key parameter in the efficiency of converting dietary ME into NE in ruminants [5]. When referring to energetic efficiencies, two aspects deserve particular attention: (1) feed intake level, and (2) associate effects between feeds on feed digestibility (DE values). Thus, digestibility of feeds in ruminants is usually depressed as the feed intake increases. This fact has been considered in the UK feeding system [2,22] and it is taken into account through the calculation of feed units (FU) as described in the French system [5,34,35] by the gut fill effect. Using ME units when rationing cattle depends on the accurate evaluation of the ME of feed ingredients [2,12]. Differences in true feeding value of forages and concentrates tend to vary as a function of energy density of the diet and are generally reflected either in diet digestibility or metabolisability [36].

### 3.3. Energy Requirements for Growing Cattle

#### 3.3.1. Maintenance Requirements

According to Ferrell and Jenkins [37] up to 65–70% of the total energy required for meat production is used for maintenance. Therefore, accurate determination of energy requirement for maintenance plays a major role on the efficiency of utilisation of dietary energy. Energy requirement for maintenance is not constant and varies with live weight and metabolic body size as the result of animal-related (i.e., age, breed, sex, level of production, etc.) and environmental factors involved [37,38]. The equations for calculating net energy requirements for maintenance (NE_m_) for growing animals are presented in Table A2 in the Appendix A.

All models are built from the LW of the animals, either as metabolic LW (LW^0.75^) in systems based on calorimetry [2,5,12]) or further adjustment to empty body weight (EBW) in systems based on comparative slaughter [13,14]. It is interesting to note that in the equation of AFRC [2], LW is raised to 0.67 power, whereas in both equations proposed by CSIRO [12] and INRA [5], the coefficient of 0.75 is used instead. Fasting metabolism data taken from both beef and dry dairy cows in the AFRC [2], might end up with the conclusion of adopting a different power for metabolic weight. Both AFRC [2] and CSIRO [12] equations separate the energy requirements for maintenance into requirements for fasting metabolism (first term) and AFRC [2] adds an additional energy cost of activity while CSIRO [12] allows for an increased maintenance requirement as the feed intake increases by adding the factor (0.1 ME_p_ × *k_m_*; see Table A2 and Table A4) to the basal metabolic rate [12]. However, when using an earlier BR-CORTE database, Marcondes et al. [39] did not find a clear relationship between *k_m_* and the ME concentration in the diet of animals offered low digestibility feeds in tropical conditions.

Both AFRC [2] and CSIRO [12] systems recognise a higher metabolic rate for bulls when compared with steers and heifers by adding a correction factor of 1.15. The Australian system [12], makes a further adjustment by adding a correction factor to indicate the breed differences (1.2 for *Bos indicus*, and 1.4 for *Bos taurus*, respectively). In the equation proposed by CSIRO [12], the effect of age (years) is explicitly incorporated as a power of one of the equation terms. The energy requirement for maintenance in the NE systems [5,13,14] is assumed to be constant per kg of LW^0.75^ (Table A2). The French system [5] makes a distinction between pre-ruminant and ruminant animals by using different coefficients (0.289 and 0.423 for the first and second respectively). The NASEM system [13] further adjusts for the effect of environmental temperature on the metabolic rate. It is assumed a thermoneutrality of 20 °C and adjusts for either cold or heat stress.

**Figure 1:**
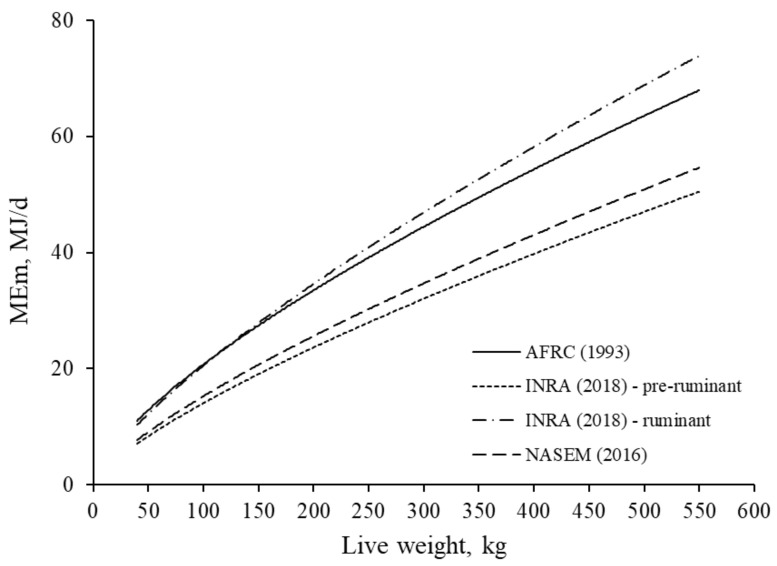
The relationships between LW and energy requirement for maintenance calculated for growing *Bos taurus* bulls according to equations by AFRC [2], INRA [5], and NASEM [13]. For comparison purposes among energy systems, a constant *k_m_* value was assumed to be equal to 0.65.

The relationships between LW of *Bos taurus* bulls and ME_m_ calculated from equations within three energy systems [2,5,13] are presented in Figure 1. For the simulation using the equation by NASEM [13], calculations were done assuming thermoneutrality. Both CSIRO [12] and BR-Corte [14] equations were discarded for this comparison between energy systems. The reasons for this include: 1) In the equation by CSIRO [12], growth curve data is required for calculations and ME for production is assumed no to be constant even at the same q value [12], and 2) In the equation by the Brazilian system [14] was also discarded because it was developed based on both Zebu and Zebu crossbred’s data which is not representative of the UK conditions. Moreover, in the BR-Corte system there is not a specific equation for pure *Bos taurus* animals. In that system, only an equation for crossbred (*Bos taurus × Bos indicus*) animals is available.

The NRC [19] suggests that *Bos indicus* would have 10% less NE_m_ when compared to *Bos taurus* animals. The NE_m_ requirement in these three energy systems is a curvilinear function that is reduced per kg of metabolic weight (LW^0.75^) with increasing LW of cattle. Overall, the maintenance requirement (ME_m_) calculated by using INRA [5] (ruminant equation) was higher than ME_m_ outputs from AFRC [2] and NASEM [13] respectively. However, relationships were rather similar for LW of less than 180 kg (pre-weaning) for both European systems. Interestingly, INRA [5] equation for pre-ruminant animals, although yielding slightly lower ME_m_, is in close agreement with outputs given by the NASEM [13].

**Figure 2:**
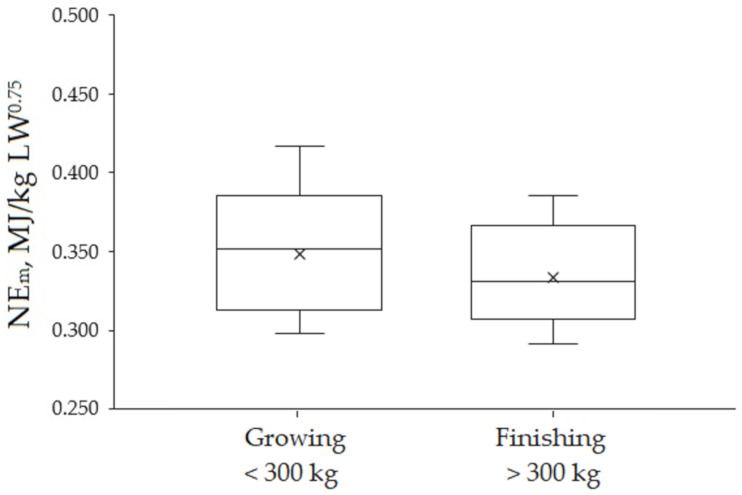
Boxplots of energy requirements for maintenance (NE_m_) summarizing data from studies conducted with Zebu and crossbred cattle in Brazilian conditions. The values reported in the following studies were taken for obtaining this plot: Growing: [42,43,44,45,46,47,48]; Finishing: [44,45,46,48,49,50,51].

Calorimetry studies conducted at AFBI with *Bos taurus* genotypes revealed 21% increased ME_m_ requirements for growing animals when compared to finishing animals (0.781 vs. 0.617 MJ/kg LW^0.75^). The last comparison for the studies by Jiao et al. [40] and average of studies by Gordon et al. [41], and Dawson and Steen [38], respectively (Table 2). The effect of the physiological state on NE_m_ requirement in growing animals from comparative slaughter studies in Brazilian conditions is illustrated in Figure 2. Finishing bulls had a 4.3% lower NE_m_ requirements than the observed in growing animals below 300 kg LW (0.334 ± 0.0335 vs. 0.349 ± 0.0420 MJ of NE_m_/ kg LW^0.75^ for finishing and growing animals respectively; *p* = 0.426). Further comparisons such as measurement technique, breed type and gender were not carried out in the present review because of the paucity of data.

In the data compiled earlier by Cottrill et al. [1], with a higher participation of *Bos taurus* animals within the dataset, the NE_m_ requirements were slightly greater when compared with most recent data collected in the present review which was derived mostly from comparative slaughter trials with Zebu animals and their crossbred’s (0.353 vs. 0.336 MJ/kg LW^0.75^ respectively; see Table 2). Discrepancies in ME_m_ values among studies may come from differences at individual animal basis in converting the ME into NE for maintenance and differences in the method of calculations of *k_m_*. Considering a hypothetical example of a *Bos taurus* bull (LW = 300 kg) and a fixed *k_m_* value of 0.65, the estimated ME_m_ requirement according to AFRC [2] and NASEM [13] equations appear to be 8.2% and 19.5% lower respectively when compared to the mean value of 0.672 MJ/kg^0.75^ obtained from calorimetry studies conducted at AFBI (Table 2). However, this is in close agreement with the prediction given by the INRA [5] equation for ruminant animals (0.671 MJ/kg^0.75^; see Table A2 for further details). Data from both Jiao et al. [40] and present literature review are in line with findings by Cottrill et al. [1] that there is a of lack of evidence to support 1.15 times increased ME_m_ requirements for maintenance for bulls compared with steers and heifers, as recommended by the ME systems [2,12] (Table A2).

The selection of individuals on a residual feed intake (RFI) basis has been subjected to an extensive research over the last decade worldwide and the effects of such strategy on their maintenance requirements of energy already dilucidated at some extent for growing animals in Irish conditions. The feeding study conducted by Lawrence et al. [52] with growing Simmental × Holstein-Friesian heifers predetermined according to a phenotypic RFI classification in Irish conditions, estimated that NE_m_ requirements calculated from regressing daily LW gain (g/kg LW^0.75^) against NE intake were equivalent to 0.410, 0.368, 0.335 MJ of NE_m_/kg LW^0.75^ for the high, medium, and low RFI groups respectively (LW = 311 kg at the beginning of the test period). These values are in line with those measured in Nellore steers by Gomes et al. [53] (Table 2), where the high RFI animals displayed 18% increased ME_m_ requirements when compared to low RFI animals (0.778 vs 0.637 MJ of ME_m_ /kg LW^0.75^ respectively for the high and RFI groups). The NE_m_ values obtained by Lawrence et al. [52] were not included in Table 2 since energy metabolism calculations were not in the main objectives of that study.

#### 3.3.2. Live Weight Gain

The equations proposed by the energy systems to estimate NE requirement for LW gain (NE_g_) for growing cattle are presented in Table A3 in Appendix A. Both LW and daily LW gain provide the basis of the NE_g_ calculations for the AFRC [2], CSIRO [12], NASEM [13] and BR-Corte [14]. The AFRC [2] includes adjustments to the NE_g_ equation depending on animal gender (bull, castrate or heifer) and breed type (early, medium or late maturing). The correction factor (CF) is highest for an early maturing (e.g., Aberdeen Angus) heifer (CF = 1.30) and lowest for a late maturing breed (e.g., Charolais) bull (CF = 0.70). The French system [5] takes into account the amounts of protein and lipids retained aiming for more accurate NE_g_ predictions compared to energy systems based only in LW measures. In the past, observable body composition (fat and protein) differences among cattle breeds have resulted in studies suggesting variation in body composition to be a major driver in fasting or maintenance energy expenditure [37]. Nowadays, this approach might be justified by non-invasive methods such as: computed tomography, ultrasound, etc., enabling more frequent measures with high accuracy [56]. Although INRA [5] has developed specific equations for estimating the daily accretion of body molecules (lipid and protein), there is still a question mark on the applicability of this approach in farm conditions. Nevertheless, the composition of empty body gain (EBG) is the main driver of energy requirements for LW gain, which is estimated from retained energy in the body. What determines the composition of EBG is not the absolute body weight, but the weight relative to animal maturity [14,57]. The calorimetry study by Posada-Ochoa et al. [45] conducted with Nellore bulls (Table 2), strongly indicates that as the animal gets heavier, the energy requirement for maintenance decreases per kg of LW^0.75^. Earlier studies have suggested that it can be partly explained by the lower weight proportion of organs and body protein as age increases [58]. However, Posada-Ochoa et al. [45] did not find significant differences either on *k_m_* values estimated from linear regression between heat production (HP) and ME intake (MEI) at ad libitum and maintenance feeding levels or in *k_g_* values.

The American system [13] estimates the NE requirements for gain (NE_g_) from the empty body weight and from the desired empty body weight gain. This equation was built considering a steer weighing 478 kg and with a body fat content of 28%. The NASEM [13] and its former NRC version [19] still recommends applying the 18% factor for more or for less to obtain the net energy requirements for weight gain of heifers and bulls, respectively. A higher growth ability for bulls compared to heifers reared on a high-forage diet was documented in the early work by Steen [4]. Responses in lean gain to increasing feed intake (per MJ of ME) were 2.5 and 1.5 times greater in bulls and steers, respectively, than in females despite the energy supply.

**Figure 3:**
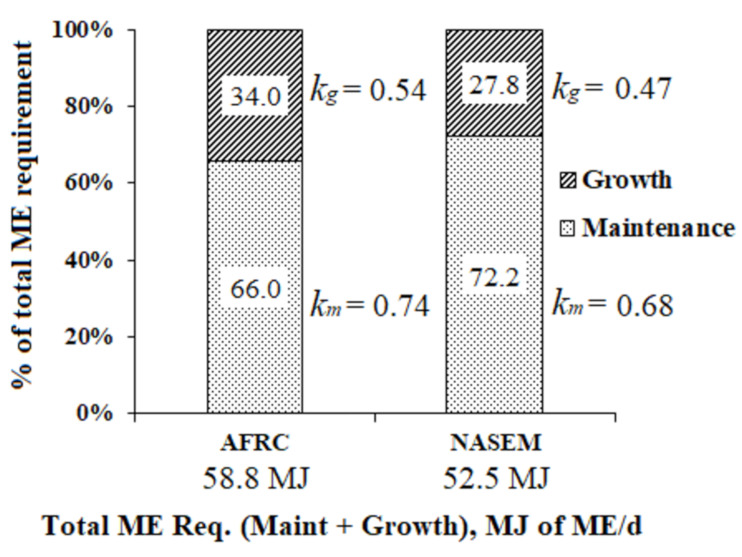
Simulation of total ME requirement calculated by equations within two feeding systems: AFRC [2], and NASEM [13] by taking an example provided by Gordon et al. [41]. Observed MEI = 0.81 MJ/kg^0.75^ (total MEI = 74.6 MJ/d).

The sum of energy requirements for both maintenance and growth is assumed to be the total requirement for a growing animal. For comparison purposes on the implications of using energy requirements for maintenance and growth by using equations within either the AFRC [2] or the NASEM system [13], a hypothetical example was taken from the study conducted in AFBI by Gordon et al. [41], where the total energy requirements of an Angus × Friesian steer (LW= 416 kg) offered good quality grass silage only (11.5 MJ of ME per kg of DM) were calculated (Figure 3). A 5% safety margin was added to ME requirements calculated from equations of AFRC [2] as recommended in their guidelines. In this example, the total energy requirement (MJ of ME/d) as predicted by the AFRC [2] and the NASEM [13] equations represented 78.8 and 70.4% of the observed MEI (74.6 MJ/d). Although the energy requirement for maintenance related to the total ME requirement is proportionally greater when using the North American standard, both systems substantially underpredict ME_m_ in (23.8 and 37.5 % for the AFRC and NASEM respectively) when compared to the measured ME_m_ value in the referred study (0.620 MJ of ME/ kg LW^0.75^). In the study by Gordon et al. [41], the *k_g_* was calculated to be 0.39 by regression analysis. Both feeding systems overestimate the efficiency of converting ME for growth (*k_g_* = 0.54 and 0.47 for the AFRC and NASEM systems respectively). The effect of equations for calculating diet metabolisability (ME/GE; see Table A1) on the efficiency of energy utilisation predictions (*k*’s), especially for *k_g_* may have contributed to enlarge these differences reflecting on total ME requirements. The last may support the inclusion of the accretion rates of lipids and protein in muscle for a better estimation of NE_g_ for growing animals in Northern Ireland conditions as considered by the French system [5] (not included in this simulation as carcass composition data was not available in the original publication). As the efficiencies of energy utilisation (*k*’s) rely on diet metabolisability in the AFRC system [2], a re-evaluation of this concept is recommended towards updating the British system.

Compensatory growth involves an upward shift in the efficiency of use of ME energy for LW gain [57]. The partial efficiency of the use of metabolisable energy for gain (*k_g_*) and the EBW affected the *k_m_*, which suggests that the maintenance requirements are affected by the performance of the animals [59]. The first cause of compensatory growth by immature animals given abundant feed after a period of undernutrition is probably an above-average feed intake [12]. These growth responses are of particular interest for beef cattle raised under grazing conditions, where animals do not always have sufficient food available at particular times over the year. However, these may not occur immediately after changing to a plentiful supply of food. The UK system [2] does not take into account compensatory growth responses within calculations of energy requirements. The Australian system [12] accounts for compensatory growth of immature animals after a period of sub-optimal nutrition. The recently updated French system [5] did not account for the influence of compensatory growth response on energy requirements although acknowledge the necessity of mechanistic approaches for dealing with this. According to NRC [19], the ME_m_ requirement decreases up to 20% in animals experiencing compensatory growth. As a result, this increases the energy availability for LW gain at the same energy intake with this low requirement associated with smaller size of internal organs due to the feed restriction. Net energy for LW gain is also reduced in up to 18% indicating improved energy efficiency in compensating animals [60]. According to the same authors, compensatory gain can be attributed to gut fill and increased tissue gut weight and other internal organs. Animal responses in compensatory growth may also vary depending on age. Drouillard et al. [61] maintained steers in growing phase under energy and protein restriction for 77 days. The authors observed that compensatory growth was similar regardless of energy or protein restriction. Moreover, at the finishing phase animals under energy restriction maintained better performance when compared with those under protein restriction, where restriction of both energy and protein supply was longer. Compensatory growth is an intrinsic part of grass-based beef production systems in Ireland. Growing animals are usually offered forage-based diets of moderate nutritive value (i.e., nutrient restriction) over the more expensive indoor winter period (store period), which usually results in compensatory growth subsequently when grazing more cheaply produced, higher nutritive value grass [62]. Studies aiming to account for the effect of compensatory growth responses on energy requirements in beef cattle are particularly lacking in the UK and Ireland.

### 3.4. Energy Requirements for Suckler Cows

Although considerable data on the energetics of specialised dairy-type cows have been reported, data for lactating beef cows are particularly lacking. One of main the reasons explaining this is the inherent difficulty to accurately estimate milk production in a cow that is suckled by a calf [63].

#### 3.4.1. Maintenance Requirements

Equations for calculating net energy requirements for maintenance (NE_m_) for suckler cows are presented in Table A4. Although principles for calculations are the same as described in the section for growing animals, equations terms mostly differ when compared to the latter animals in the AFRC [2], CSIRO [12], and INRA [5]; whereas equations are the same as for the growing animals in both NASEM [13] and BR-Corte [14]. The French model [5], recognise different maintenance requirements for gestating and lactating animals, suggesting lower energy requirements for dry cows as reported in earlier studies [64]. This might be expected due to dry cows generate less metabolic heat when compared to energy demands related to milk production in lactating cows.

**Figure 4:**
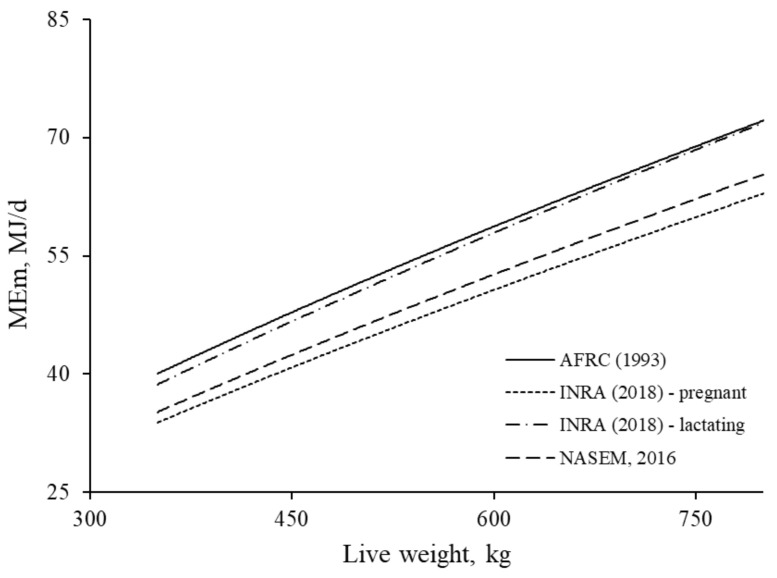
The relationships between LW and energy requirement for maintenance calculated for *Bos taurus* suckler cows according to equations by AFRC [2], INRA [5], and NASEM [13]. For comparison purposes among energy systems, a constant *k_m_* value was assumed to be equal to 0.72.

The relationships between LW of *Bos taurus* suckler cows and ME_m_ calculated from equations within the energy systems [2,5,13] are presented in Figure 4. The ME_m_ requirements for suckler cows according to AFRC [2] are comparable with those from INRA [5] for lactating cows, whereas requirements by NASEM [13] are slightly greater than those according to INRA [5] model for pregnant cows.

Literature values on energy requirement for the maintenance (NE_m_ and ME_m_) of suckler cows are presented in Table 3. There is a wide range of ME_m_ values published in the literature for lactating cows irrespective of the technique used to estimate ME_m_. Compared to growing cattle, fewer studies were found in the literature estimating maintenance requirements in suckler cows. The ME_m_ values for suckler cows ranged from 0.389 to 0.796, a mean of 0.596 (s.d. 0.1580) MJ/kg^0.75^. This mean value is considerably greater than the ME_m_ requirement predicted by the energy systems per unit of metabolic weight (assuming a cow of 550 kg of LW and a *k_m_* value of 0.72) of: 0.486 MJ/kg^0.75^ according to the AFRC [2]; 0.418 and 0.478 MJ/kg^0.75^ for pregnant and lactating cows respectively in the INRA system [5], 0.434 MJ/kg^0.75^ as calculated by the NASEM equation [13], and 0.388 MJ/kg^0.75^ according to BR-Corte [14]. The mean ME_m_ value found in the present review (which is based on literature data since 2009), is in line with the 0.583 MJ/kg^0.75^ calculated from the studies compiled earlier by Cottrill et al. [1] for the period of 1989–2009. In specialised beef-type breeds, an underestimation of the energy requirement for maintenance has been reported as high as 30% for suckler cows [64]. Studies that aim to determine the energy requirements of suckler cows are particularly lacking in the UK conditions (Table 3; only one study conducted by Zou et al. [65]). In that study, the authors did not find a significant effect of suckler cow genotype on energy intakes, energy outputs or energy use efficiency despite Holstein Friesian cows have a greater milk production potential than Stabiliser cows. Most likely, stage of lactation may have influenced these responses since these cows were non-lactating and in the last 100 days of pregnancy and this may have had an effect on energy utilisation and performance.

On a number of occasions, the recommendations of energy requirements for maintenance in suckler cows in energy systems worldwide have been focused on specialised dairy type breeds, as there is relatively much less research conducted with suckler cows. In the case of updating the UK recommendations for beef cattle [2,22], there is still a question mark on the applicability FIM [27] equations for predicting the energy requirements for suckler cows.

#### 3.4.2. Pregnancy

Equations for calculating net energy requirements for pregnancy (NE_gest_) for suckler cows are presented in Table A5 in the Appendix A. The energy cost of gestation includes the growth and maintenance of uterine, mammary and other tissues, the maintenance of the foetus [2,5,13] and any augmentation of maternal metabolism is expressed as a function of gain by the conceptus only [12]. The pregnancy requirements and weight gain from the growth of the gravid uterus are based on expected calf birth weight and day of gestation are the basis for energy calculations. The efficiency of using ME for conceptus energy gain did not differ greatly across energy systems. Feeding systems based their recommendations to meet pregnant suckler cow nutritional requirements on a few studies carried out long ago or on indirect estimates and adaptations of values obtained in experiments involving other ruminant categories or species. For the UK system, the ARC [3] based their recommendations in a study involving Ayrshire and Jersey cows carried out in 1975 and the AFRC [2] did not provide a significant update on how to calculate nutritional requirements for pregnancy.

#### 3.4.3. Lactation

The equations to calculate energy contents in milk for lactating in suckler cows (NE_l_) are presented in Table A6 in the Appendix A. All energy systems [2,5,12,13] use concentrations of milk components (fat, protein and lactose) to estimate the energy value of milk and thus estimate the energy requirement for lactation (NE_l_). Depending on availability of milk composition data, the AFRC ([2], recommend using one of the three equations proposed by Tyrell and Reid [70], based either on: fat, fat and protein, or fat, protein and lactose contents to estimate energy value of milk (EVM). Furthermore, EVM is multiplied with milk yield, and the obtained value divided by the calculated lactation efficiency (*k_l_*). The use of all three component concentrations in milk to estimate NE_l_ is recommended rather than using fat concentration only because a higher accuracy on energy contents in milk might be expected.

**Figure 5:**
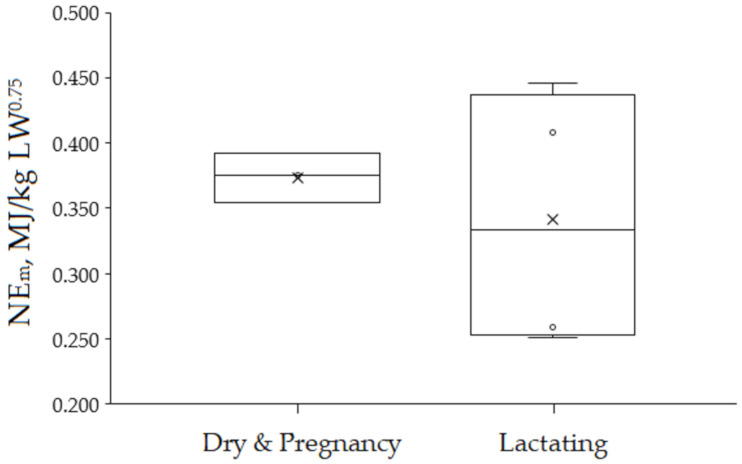
Boxplots of energy requirements for maintenance (NE_m_) summarizing data from studies conducted with suckler cows at two physiological stages (mostly *Bos taurus* animals). The values reported in the following studies were taken for obtaining this plot: dry and pregnant cows [65,69]; lactating cows: [29,67].

The effect of the physiological state on NE_m_ requirement in suckler cows from data collected in the present study is illustrated in Figure 5. For this comparison, both experiments by Wiseman et al. [66] and Trubenbach et al. [30] were discarded. This was because limit-fed lactating may compromise normal lactation performance of primiparous cows [66] and requirements were not reported on a NE_m_ basis in [30]. In this comparison the NE_m_ requirement did not differ significatively between physiological stages (0.374 ± 0.0190 vs. 0.341 ± 0.1006 MJ/kg LW^0.75^ for dry and pregnant, and lactating cows respectively, *p* = 0.611). However, the observed NE_m_ for lactating animals displayed much larger variation (c.v. = 29.5%) than the one for the pregnant animals (c.v. = 5.1%). Earlier studies reported a range of 10 to 27% increase in maintenance energy requirements for lactating cows using a constant diet ME value [64], and a 16% increase in maintenance energy requirements for pregnant, lactating Angus × Hereford dams compared with non-pregnant, non-lactating Angus × Hereford cows [37]. These studies agree with the predictions given by equations within the INRA system [5] as presented in Figure 5. Nevertheless, the NE_m_ values summarized in Figure 5 should be treated with caution, due to the few number of studies included and quite large differences in experimental conditions within individual studies.

## 4. Conclusions

The present review confirmed previous reports on underprediction of energy requirements for maintenance (ME_m_, and NE_m_) for both growing animals and suckler cows when using the AFRC system [2]. This may have consequences on the predictions of energy partition for productive functions as demonstrated in the present review by a simulation performed for an Angus × Friesian steer (LW = 416 kg) offered good quality grass silage, where an overprediction of *k_g_* was found (observed *k_g_* = 0.39 vs. predicted by AFRC *k_g_* = 0.54). Overall, suckler cows’ data on energy requirements for maintenance collected from recent studies in the literature (2009–2020) appears to be more representative to be used as a reference for updating UK energy models than compiled data for growing animals which was mostly derived from studies conducted with Zebu cattle and their crossbreds in Brazilian conditions. Among feeding systems, predictions of energy requirements given by the INRA model [5] seem to be more realistic for application in UK conditions compared to other models.

## Figures and Tables

**Table 1:** General descriptors of international nutritional models on determination of energy requirements for beef cattle ^1^.

Nation of Origin	Organisation	Date	Publication Name	Animal Type	Maint. Req.	Energy Units ^1^	CH_4_ Equations	Observations
UK	Agriculture and Food Research Council (AFRC, [2]), formerly Agriculture Research Council (ARC, [3,22])	1993	Energy and Protein Requirements of Ruminants	Continental and British breeds	Calorimetry	ME	No	Still provides an important theoretical background for the majority of energy systems worldwide. Forage-based diets.
Australia	Commonwealth Scientific and Industrial Research Organisation (CSIRO, [12])	2007	Nutrient Requirements of Domesticated Ruminants	*Bos taurus*, *Bos indicus* and crossbreds	Calorimetry	ME	No	CSIRO guidelines follow the AFRC approach to use of ME_m_ as the measure of maintenance requirements. Feed tables include low quality forages.
France	Institut National de la Recherche Agronomique (INRA, [5,28,29])	2018	INRA Feeding System for Ruminants	Beef and dairy origin genotypes	Calorimetry	NE	Yes	NE is expressed in terms of barley feed unit (1 FU = 1760 kcal for 1 kg of fresh standard barley).
USA and Canada	National Academies of Sciences, Engineering and Medicine (NASEM, [13]). Update on National Research Council (NRC, [19]) guidelines	2016	Nutrient Requirements of Beef Cattle. 8th revised edition 2016	*Bos taurus*, *Bos indicus* and crossbreds	Comparative slaughter	NE	Yes	North America diets for feeding beef cattle typically contain high concentrate levels compared to other countries. NASEM (2016) provides levels of solutions from empirical to more mechanistic approaches.
	Ruminant Nutrition System (RNS, [6]) Project	2018	The Ruminant Nutrition System. 2nd edition	*Bos taurus*, *Bos indicus* and crossbreds	Comparative slaughter	NE	Yes	The RNS is a further development of the Cornell Net Carbohydrate and Protein System published during the 2000s decade. The RNS includes 3 levels of solutions (L0, L1, and L2) from empirical to more mechanistic approaches.
Brazil	Universidade Federal de Viçosa (UFV) (BR-Corte, [14])	2016	BR-Corte 3rd edition	Zebu cattle and crossbreds	Comparative slaughter	NE	Yes	Zebu cattle is mainly Nellore. Energy equations for both feedlot and pasture conditions. Calorimetry was recently introduced to estimate energy requirements.

^1^ For comparison purposes, energy systems can be grouped in two main categories. 1. metabolisable energy (ME) systems, which includes AFRC [2,3,22] and CSIRO [12] and 2. net energy (NE) systems, which includes French (INRA) [5], North America (NRC, NASEM, and RNS) [6,13,19], and Brazilian (BR-Corte) [14] systems. In both UK and Australian systems, units for energy equations are in Mega Joules (MJ), whereas systems in North America and Brazil, calories are preferred. Feed units in the French system [5] are usually converted to calories equivalent. One calorie = 4.184 MJ.

**Table 2:** Metabolisable and NE requirements for maintenance for growing beef cattle from recent studies published around the world ^1^.

**Reference**	**Country**	**Technique**	**Anim.**	**Type**	**Breed**	**LW (kg)**	**ME_m_ (MJ/kg LW^0.75^)**	**NE_m_ (MJ/kg LW^0.75^)**
**AFBI studies ^2^ (1990–2020)**								
Jiao et al. [40]	UK	Calorimetry	20	Steers, heifers	Holstein	176	0.781	0.570
Gordon et al. [41]	UK	Calorimetry	12	Steers	Angus × Friesian	416	0.620	--
Dawson and Steen [38]	UK	Calorimetry	75	Steers	Beef cross	450–628	0.614	--
**International studies ^3^ (2009–2020)**								
Castro et al. [42] *	Brazil	Comp. laughter	22	Heifers	Holstein × Gyr	98–172	0.545	0.352
Ferreira et al. [49] *	Brazil	Calorimetry	15	Bulls	Holstein × Gyr	302	0.523	0.312
Silva et al. [43]	Brazil	Comp. Slaughter	39	Bulls	Holstein × Gyr	43–93	--	0.298
Oss et al. [44]	Brazil	Comp. slaughter	24	Bulls	Holstein × Gyr	182–388	--	0.313
Posada-Ochoa et al. [45] *	Brazil	Calorimetry	5	Bulls	Nellore	219	0.691	0.418
	Brazil	Calorimetry	5	Bulls	Nellore	328	0.567	0.332
	Brazil	Calorimetry	5	Bulls	Nellore	394	0.512	0.331
	Brazil	Calorimetry	5	Bulls	Nellore	473	0.468	0.303
Salah et al. [31]—Meta-analysis	France	Feeding studies	1855	Growing animals	Temperate and tropical phenotypes.	--	0.631	--
Marcondes et al. [46]—Meta-analysis*	Brazil	Comp. slaughter	752	Growing animals	Nellore, Nellore × *Bos taurus*	258–426	--	0.386
Rotta et al. [51] *	Brazil	Comp. slaughter	44	Bulls	Holstein × Zebu	338	0.555	0.382
Sainz et al. [50]—Meta-analysis*	USA	Comp. slaughter	127	Steers	Angus, Hereford and crossbreds	--	--	0.314
	Brazil	Comp. slaughter	711	Bulls	*Bos indicus*	--	--	0.292
Valente et al. [47] *	Brazil	Comp. slaughter	46	Bulls	Nellore	138	0.603	0.325
Gomes et al. [53] *	Brazil	Comp. slaughter	8	Steers	Nellore, High RFI	340–348	0.778	--
	Brazil	Comp. slaughter	9	Steers	Nellore, Low RFI	334–441	0.637	--
Porto et al. [48]	Brazil	Comp. slaughter	10	Bulls	Nellore × Holstein	199–317	0.607	0.352
**Summaries ^4^**								
AFBI studies (1990–2020)							0.672 ± 0.0947	0.570
Literature (2009–2020)							0.593 ± 0.0846	0.336 ± 0.0372
Cottrill et al. [1]—Review (1989–2009) ^5^							0.524 ± 0.0776	0.353 ± 0.0775

^1^ Source: CAB Abstracts, Web of Science, ISI Proceedings, BIOSIS Previews, Food Science and Technology Abstracts and MEDLINE; ^2^ AFBI studies (1990–2020). Three publications. The study by Dawson and Steen [38] collated experimental data from Kirkpatrick et al. [54], Kirkpatrick [55], and Lavery and Steen (unpublished data); ^3^ International literature (2009–2020). Twelve publications. The following adjustment was made to obtain energy requirements in terms of LW units: EBW = (0.861 ± 0.0031) × LW [44]. * Estimated requirements from EBW units (empty body weight). In the calorimetry study by Posada-Ochoa et al. [45], the same five Nellore bulls were used to calculate energy requirements for maintenance at four periods (LW targets) during the growing-finishing period; The meta-analysis by Marcondes et al. [46] included: 431 bulls, 204 steers and 117 heifers; In the study by Gomes et al. [53], RFI = residual feed intake; ^4^ Summaries are mean and s.d. values of energy requirements for maintenance.^5^ The review by Cottrill et al. [1] includes eleven publications excluding data from AFBI studies: Gordon et al. [41], and Dawson and Steen [38]. In Cottrill et al. [1] report there was a greater participation of *Bos taurus* genotypes (4 publications) when compared with the updated review of international literature (2009–2020) compiled in the present review.

**Table 3:** Metabolisable and NE requirements for maintenance for suckler cows from recent studies published around the world.

References	Country	Technique	Animals	Breed	Physiological State	LW (kg)	ME_m_ (MJ/kg LW^0.75^)	NE_m_ (MJ/kg LW^0.75^)
Andresen et al. [29]	USA	Feeding studies	32	Aberdeen Angus	Milking cows	505–516	0.389	0.251
		Feeding studies	27	Hereford × Angus	Milking cows	518–516	0.400	0.259
Trubenbach et al. [30]	USA	Feeding studies	31	Angus × Nellore	Milking cows	433–477	0.736	--
Wiseman et al. [66]	USA	Feeding studies	45	Angus and Angus × Hereford	Trad. weaning, 226 d	417–445	0.471	0.288
		Feeding studies	45		Early weaning, 130 d	414–445	0.447	0.274
Carvalho et al. [67]	Brazil	Calorimetry	6	Gyr	Milking cows	483	0.729	0.408
		Calorimetry	6	Gyr × Holstein	Milking cows	510	0.796	0.446
Zou et al. [65]	UK	Calorimetry	17	Limousin × Holstein Friesian	Dry & pregnancy	589	0.728	0.392
		Calorimetry	17	Stabiliser *	Dry & pregnancy	679	0.697	0.375
Fiems et al. [68]	Belgium	Feeding studies	60	Belgian Blue	Dry & non pregnant	--	0.569	0.332
Cooper-Prado et al. [69]	USA	Feeding studies	93	Aberdeen Angus	Dry & pregnancy	582	--	0.373
**Summaries**								
Present review (2009–2014)							0.596 ± 0.1580	0.340 ± 0.0687
Cottrill et al. [1]—Review (1989–2009)							0.583 ± 0.0605	N.A.

Source: CAB Abstracts, Web of Science, ISI Proceedings, BIOSIS Previews, Food Science and Technology Abstracts and MEDLINE. International literature (2009–2020). In total seven publications including the AFBI study by Zou et al. [65]. * Stabiliser = is a composite breed of cattle developed in America by Lee Leachman of Colorado (www.leachman.com, accessed on: 7 December 2020). The study by Wiseman et al. [66] was conducted in primiparous cows. Cottrill et al. [1] Review (1989–2009) includes five publications involving calorimetry, comparative slaughter and long-term feeding studies. In that earlier review, two studies included Zebu and Zebu × *Bos taurus* crossbreds. N.A.= Not available.

## Data Availability

The data presented in this review are available on request from the corresponding authors.

## Appendix A

**Table A1:** Equations used to calculate efficiencies of ME utilisation for maintenance, growth and lactation in global energy system and calculated values at two diet metabolisability values.

System	Equation, *k*_s_	ME/GE *		Reference
		0.50	0.65	
AFRC (1993); CSIRO (2007)	*k_m_* = 0.35 ME/GE + 0.503	0.68	0.73	[2,12]
	*k_g_* = 0.78 ME/GE + 0.006	0.40	0.51	
	*k_l_* = 0.35 ME/GE + 0.42	0.60	0.65	
INRA (2018)	*k_m_* = 0.287 ME/GE + 0.554	0.70	0.74	[5]
	*k_f_* = 0.78 ME/GE + 0.006	0.40	0.51	
	*k_mf_ =* (*k_m_* × *k_f_* × 1.5)/(*k_f_* + 0.5 × *k_m_*)	--	--	
	*k_pf_* = 0.35 + 0.25 × (1 − *EP*)^2^	--	--	
	*k_ls_* = 0.65 + 0.247 (ME/GE *−* 0.63)	0.62	0.65	
NASEM (2016)	*k_m_* = (1.37 ME − 0.138 ME^2^ + 0.0105 ME^3^ − 1.12)/ME	0.61	0.67	[13]
	*k_g_* = (1.42 ME − 0.174 ME^2^ + 0.0122 ME^3^ − 1.65)/ME	0.35	0.45	
BR-Corte (2016)	*k_m_* = [(0.513 + 0.173 × *k_g_* + β_2_ × EBG) × θ]	--	--	[14]
	*k_g_* = 0.327/(0.539 *−* REp)	--	--	

* ME/GE is metabolisability. CSIRO [12]. Although the Australian system largely adopted the principles and equations used in the AFRC [2], those equations were converted in terms of M/D (MJ of ME per kg of feed DM). There is a specific equation for *k_g_* in grazing conditions. *k_g_* = 0.035 M/D (1 + 0.33 Le) (1.0 + 0.12(λ sin (0.0172T)/40)); where: Le = the proportion of legume in the forage, T = the day of the year from 1 January, h = the latitude (°) of the site; negative in the south. Otherwise, *k_g_* in Table A1 (converted to M/D equivalents), is recommended for concentrate and grass silage-based diets; INRA [5]. The ME units= Mcal/kg of DM; EP= protein proportion in LW gain. EP = 5.48 ProtGain/(5.48 ProtGain + 9.39 LipGain); For slow growing cattle (LW gain ≤ 1 kg/d; a metabolisability coefficient is calculated instead as: qprimma = 0.62 − 0.262 × exp(−3.175 × LW gain), with LW gain in kg/d; *k_f_* = fattening; *k_mf_* = combined efficiency of ME for maintenance, growth and meat deposition for fast-growing animals [29]; *k_pf_* = protein and fat deposition (known body gain composition); *k_ls_* = milk yield + maintenance for lactating animals/ maintenance and gain for slow-growing cattle. Both *k_g_* and *k_l_* as such are used as such in the up-to-date version of the French system [5]. NASEM [13], same equations for *k_m_* and *k_g_* as in the NRC [19]. Values for energy efficiencies (*k_s_*) are not based on diet metabolisability (ME/GE); BR-Corte [14], the efficiency for maintenance includes *k_g_*; EBG = empty body gain (kg/d), β_2_ = 0.100 for Zebu, 0.073 for beef crossbred and 0.010 for dairy crossbred and θ = fit factor for the rearing system that takes the value of 1 for animals reared on feedlot, and 0.92 for pasture reared animals. There is not explicit mention on *k_l_* calculations. Rather than providing information to estimate energetic efficiencies, the NASEM [13], included equations to calculate dietary NE concentrations for maintenance and LW gain. For comparison purposes, the *k_m_* and *k_g_* values in NASEM [13] are estimated by dividing NE data by ME values as proposed by Cottrill et al. [1]; see Table 2, when assuming metabolisability coefficients of 0.50 and 0.65. Conversely, in the Brazilian system [14] the principle for calculating both *k_m_* and *k_g_* values is not based on ME/GE ratio. Instead, both animal-related and production system factors are considered. The authors did not obtain accurate *k_g_* predictions based on ME concentration in the diet [14].

**Table A2:** Equations used to calculate maintenance requirements (NE_m_, MJ/d) in growing animals *.

Systems	Equations	Reference
AFRC (1993)	C (0.53 (LW/1.08)^0.67^) + 0.0071 LW	[2]
CSIRO (2007)	CKM × 0.28 LW^0.75^ e^(−0.03A)^ + 0.1 MEp × *k_m_*	[12]
INRA (2018)	0.289 LW^0.75^/0.423 LW^0.75^	[5]
NASEM (2016)	0.00293 (20 − Tp) + 0.322 SBW^0.75^	[13]
BR-Corte (2016)	0.314 × EBW^0.75^	[14]

* For comparison purposes, energy coefficients are expressed in MJ (1 Mcal = 4.184 MJ); ME_m_= NE_m_/km. In AFRC [2], C = 1.0 for females and castrates, 1.15 for males. The factor 1.08 converts LW to fasted body weight [3]; Activity allowance: 0.0071LW; LW= live weight. In CSIRO [12]. Generalized equation without excluding energy expenditure at pasture and additional energy expenditure for low temperatures. C = as in the AFRC equation; K = 1.2 for *Bos indicus*, 1.4 for *Bos taurus*; M = is the fraction of the DE intake provided by milk. For convenience where the proportion of milk in the diet is not known, M can be estimated from the following equation: M = 1 + (0.26 − B × a), where B = 0.010 is a coefficient for suckled calves and a is week of life; A = age in years; MEp = the amount of dietary ME being used directly for production. In INRA [5] equation for growing and finishing beef; NE_m_ (MJ/kg^0.75^) = 0.289 LW^0.75^, and 0.423 LW^0.75^ for pre-ruminant and ruminant animal respectively; the NE_m_ increased from 88 kcal NE/kg^0.75^ [29] to 101 kcal NE/kg^0.75^ in the updated version. The latest value was theoretically determined from feeding trials by regression techniques. In NASEM [13], Tp = ambient temperature; SBW= shrunk body weight; equation can be further adjusted by multiplying it for breed factor ranging from 0.9 (e.g., Brahman) to 1.2 (e.g., Holstein). In BR-Corte [14] the equation is valid for both feedlot and pasture conditions; EBW= empty body weight.

**Table A3:** Equations used to calculate energy requirements for LW gain (NE_g_) *.

Systems	Equations	Reference
AFRC (1993)	C (4.1 + 0.0332 LW − 0.000009 LW^2^)/(1 − C_2_ 0.1475 LWG)	[2]
CSIRO (2007)	0.92 [(6.7 + R) + (20.3 − R)/(1 + e^(−6(P − 0.4))^)]	[12]
INRA (2018)	22.9 ProtGain + 39.3 LipGain	[5]
NASEM (2016)	0.266 EBW^0.75^ × EBG^1.097^	[13]
BR-Corte (2016)	0.052 × EQEBW^0.75^ × EBG^1.062^	[14]

* For comparison purposes, energy coefficients are expressed in MJ (1 Mcal = 4.184 MJ); AFRC [2] Energy value of weight gain; C = 0.70 to 1.30 for different maturity (early, medium and late) of different animal (bull, steer and female); C_2_ = 1 when plane of nutrition, L, > 1 and = 0 when L < 1; LW = live weight; LWG = live weight gain; CSIRO [12]. Equation for immature animals (energy value of gain); 6.7 and 20.3 are coefficients expressing total energy in MJ/kg; R = adjustment for rate of gain or loss; P = live weight/standard reference weight; INRA [5]. ProtGain and LipGain are protein and lipid deposition (kg/d); ME_g_= NE_g_/*k_pf_*; NASEM [13]. Retained energy; EBW = empty body weight; EBG = empty body gain; BR-Corte [14]. EQEBW = Equivalent empty body weight. This is obtained by dividing the EBW by the weight at maturity of the respective sex/genetic group and multiply by the reference weight; EBG = empty body gain.

**Table A4:** Equations used to calculate maintenance requirements in suckler cows (NE_m_) *.

Systems	Equations	Reference
AFRC (1993)	0.53 (LW/1.08)^0.67^ + 0.0095 LW	[2]
CSIRO (2007)	KM × 0.28 LW^0.75^ e^(−0.03A)^ + 0.1 MEp × *k_m_*	[12]
INRA (2018)	0.301 LW^0.75^ dry or pregnant/0.344 LW^0.75^ lactation	[5]
NASEM (2016)	0.0293 (20 − Tp) + 0.322 SBW^0.75^	[13]
BR-Corte (2016)	0.314 × EBW^0.75^	[14]

* For comparison purposes, energy coefficients are expressed in MJ (1 Mcal = 4.184 MJ). ME_m_= NE_m_/*k_m_*.; AFRC [2]. Activity allowance for lactating cows. For pregnant and non-lactating cattle: 0.0071LW; LW= live weight; CSIRO [12]. Generalised equation without excluding energy expenditure at pasture and additional energy expenditure for low temperatures. K = 1.2 for *Bos indicus*, 1.4 for *Bos taurus*; M = 1 + 0.23 × percent diet DE from milk = 1 + 0.26 − Ba, B = 0.01 for calves; A = age in years; MEp = the amount of dietary ME being used directly for production; INRA [5]. Dry or pregnant cows: Energy required in the absence of LW changes (non-productive requirements) obtained from either 120 or 137 kcal ME/kg LW^0.75^ (0.043 and 0.049 UFL/kg LW^0.75^) for dry and pregnant and lactating cows respectively and divided by *k_m_*= 0.60. A common equation for dry, pregnant or lactating beef cows adjusts maintenance requirements according to activity, LW change, and BCS. NASEM [13]. Tp = ambient temperature; SBW = shrunk body weight. BR-Corte [14]. Equation valid for both feedlot and pasture conditions. There is no specific equation for suckler cows. EBW = empty body weight.

**Table A5:** Equations used to calculate pregnancy requirements in suckler cows (NE_gest_) *.

Systems	Equations	*k_gest_*	Reference
AFRC (1993)	log_10_(Et) = 151.665 − 151.64 exp^−0.0000576t^	0.133	[2]
	0.025W_c_ (E_t_ × 0.0201 exp^−0.0000576t^)		
CSIRO (2007)	Y = SBW exp (A − B (exp(–Ct))	0.133	[12]
	DWG = nBC exp(−Ct)Y	0.133	
INRA (2018)	0.000695 × BW_calf_ × exp ^(0.116 × WG)^	0.10–0.15	[5]
NASEM (2016)	((CBW × (0.05855 − 0.0000996 × DP) × exp^(0.03233 × DP − 0.0000275 × DP2)^)/1000	0.13	[13]
BR-Corte (2016)	(CBW × 0.000000793 × TG^3.017^)/1000	0.12	[14]

* Equations from INRA [5], NASEM [13], and BR-Corte [14] expressed in calories. (1 Mcal = 4.184 MJ); *k_gest_* = energy efficiency for gestation. Requirements in terms of ME_gest_= NE_gest_/*k_gest_*; AFRC [2]. E_t_ = energy retention in MJ at time t; t = days from conception; W_c_ = calf birth weight in kg; CSIRO [12] Y = the Gompertz equation that describes the weight or energy content of the foetus or gravid uterus at time t; SBW = scaled birth weight, A, B, C = coefficients of Gompertz equation; DWG = daily gain in energy in weight or energy content; INRA [5]. BW_calf_ = expected calf birth weight; WG = week of gestation; k_gest_ = varies slightly with diet metabolisability (average 0.13); NASEM [13]. CBW = calf birth weight; DP = days pregnant; BR-Corte [14]. TG = days pregnant.

**Table A6:** Equations to calculate energy contents in milk (MJ/kg).

Systems	Equations	Reference
AFRC (1993)	MY (38.4 Fat + 22.3 Protein + 19.9 Lactose) − 0.108)	[2]
CSIRO (2007)	MY (38.1 × Fat + 24.5 × Protein + 16.5 Lactose)	[12]
INRA (1989)	UFL_MY_ = 1.84 × MY	[5]
NASEM (2016)	MY (40.6 Fat +1.51)	[13]
BR-Corte (2016)	N.A.	[14]

MY = Milk yield; AFRC [2]. In the updated version for dairy cattle [21], requirements for maintenance and milk production (together) are derived from the Mitscherlich equation. Equation for energy contents in milk was adopted from Tyrrell and Reid [70], when all individual milk contents (fat, protein, and lactose) are available; CSIRO [12]. Equation taken from Perrin [71]; INRA [5]. UFL= ME × *k**ls*/ 1760 kcal for 1 kg of fresh standard barley, where UFL is net energy feed unit for lactation and kls is efficiency of ME for milk. Requirements in UFL equivalents for energy and proteins exported in milk are calculated using average composition of a standard milk: 780 kcal; 44 g of fat and 34 g of protein, i.e., 0.44 UFL and 51 g PDI is PDI_eff_ is fixed to 0.67. PDI stands for digested protein in the intestine; NASEM [13]. Equation by Tyrrell and Reid [70] considering fat contents in milk; BR-Corte [14]. N.A.= Not available. Instead, BR-Corte recommends using the equation provided by the NRC [19].
